# Insecticide susceptibility of *Aedes albopictus* and *Ae. aegypti* from Brazil and the Swiss-Italian border region

**DOI:** 10.1186/s13071-017-2364-5

**Published:** 2017-09-19

**Authors:** Tobias Suter, Mônica Maria Crespo, Mariana Francelino de Oliveira, Thaynan Sama Alves de Oliveira, Maria Alice Varjal de Melo-Santos, Cláudia Maria Fontes de Oliveira, Constância Flávia Junqueira Ayres, Rosângela Maria Rodrigues Barbosa, Ana Paula Araújo, Lêda Narcisa Regis, Eleonora Flacio, Lukas Engeler, Pie Müller, Maria Helena Neves Lobo Silva-Filha

**Affiliations:** 1 Department of Epidemiology and Public Health, Swiss Tropical and Public Health Institute, Socinstrasse 57, PO Box, 4002 Basel, Switzerland; 2 University of Basel, Petersplatz 1, 4003 Basel, Switzerland; 3 Avia-GIS, Risschotlei 33, 2980 Zoersel, Belgium; 4 Department of Entomology, Instituto Aggeu Magalhães-FIOCRUZ, Recife 50740-465, Brazil; 5 Laboratory of Applied Microbiology, University of Applied Sciences and Arts of Southern Switzerland, Bellinzona, Switzerland

**Keywords:** Vector control, Insecticide resistance, Biolarvicides, Insect growth regulator

## Abstract

**Background:**

*Aedes aegypti* and *Ae. albopictus* are two highly invasive mosquito species, both vectors of several viruses, including dengue, chikungunya and Zika. While *Ae. aegypti* is the primary vector in the tropics and sub-tropics, *Ae. albopictus* is increasingly under the public health watch as it has been implicated in arbovirus-transmission in more temperate regions, including continental Europe. Vector control using insecticides is the pillar of most control programmes; hence development of insecticide resistance is of great concern. As part of a Brazilian-Swiss Joint Research Programme we set out to assess whether there are any signs of existing or incipient insecticide resistance primarily against the larvicide *Bacillus thuringiensis* svar. *israelensis* (*Bti*)*,* but also against currently applied and potentially alternative insecticides in our areas, Recife (Brazil) and the Swiss-Italian border region.

**Methods:**

Following World Health Organization guidelines, dose-response curves for a range of insecticides were established for both colonized and field caught *Ae. aegypti* and *Ae. albopictus*. The larvicides included *Bti*, two of its toxins, Cry11Aa and Cry4Ba, *Lysinibacillus sphaericus,* Vectomax CG®, a formulated combination of *Bti* and *L. sphaericus*, and diflubenzuron. In addition to the larvicides, the Swiss-Italian *Ae. albopictus* populations were also tested against five adulticides (bendiocarb, dichlorodiphenyltrichloroethane, malathion, permethrin and λ-cyhalothrin).

**Results:**

Showing a similar dose-response, all mosquito populations were fully susceptible to the larvicides tested and, in particular, to *Bti* which is currently used both in Brazil and Switzerland. In addition, there were no signs of incipient resistance against *Bti* as larvae were equally susceptible to the individual toxins, Cry11Aa and Cry4Ba. The field-caught Swiss-Italian populations were susceptible to the adulticides tested but DDT mortality rates showed signs of reduced susceptibility.

**Conclusions:**

The insecticides currently used for mosquito control in Switzerland and Brazil are still effective against the target populations. The present study provides an important reference as relatively few insecticide susceptibility surveys have been carried out with *Ae. albopictus*.

**Electronic supplementary material:**

The online version of this article doi:(10.1186/s13071-017-2364-5) contains supplementary material, which is available to authorized users.

## Background

Dengue (DENV), chikungunya (CHIKV) and Zika virus (ZIKV) are mosquito-borne viruses of medical importance in most tropical regions but also emerging in more temperate regions including continental Europe. Dengue fever is the most prevalent mosquito-borne disease worldwide with an estimated 390 million cases per year [1]. In the Americas, most dengue cases have been reported from Brazil, which has been affected by several epidemics since the 1990s [2]. In 2016, around 1.5 million cases were reported from all states of the country [3]. In Brazil *Aedes aegypti* is the major vector of dengue and all four DENV serotypes are co-circulating in the country [4]. In 2014, the first autochthonous chikungunya cases have been detected in Brazil and in 2016 a total of 271,824 confirmed cases have been reported from several states, including Pernambuco [3]. In 2015, autochthonous cases of ZIKV were also reported from Brazil for the first time [5] and 125,319 cases were confirmed in 2016 [3]. All the above cases have been linked to *Ae. aegypti*, a highly competent vector of arboviruses.

In continental Europe, the most prominent example is the chikungunya outbreak in Ravenna, Italy in 2007 with over 200 confirmed cases and one death. The outbreak was linked to a transmission by the invasive mosquito *Ae. albopictus* and a single viraemic person that returned with chikungunya from India [6]. CHIKV is a mosquito-borne alphavirus indigenous to African countries, the Indian subcontinent and Southeast Asia where it causes endemic and epidemic fever outbreaks [7]. The outbreak in Italy demonstrates the vector capacity of the local *Ae. albopictus* population to transmit CHIKV. Following this outbreak, additional cases of autochthonous chikungunya were recorded in mainland France [8] as well as dengue cases in both Croatia and France (e.g. [9–11]). These outbreaks show that continental Europe is vulnerable to the transmission of “tropical” arboviruses, particularly in regions where *Ae. albopictus* and *Ae. aegypti* are present.

*Ae. aegypti* and *Ae. albopictus* are the main vectors of DENV and CHIKV worldwide. Both mosquito species have recently shown a large geographical expansion. *Ae. albopictus*, known as the Asian tiger mosquito is among the 100 of the world’s most invasive alien species [11] and is currently present in many regions in the Americas, Africa, Australia and Europe, where its presence has been reported in several European countries, including Switzerland [12–14]. In Brazil, *Ae. albopictus* was recorded for the first time in 1986 and has since spread throughout the country [15]. The rapid worldwide expansion of *Ae. albopictus* is attributed to its eggs that resist desiccation and may undergo diapause, an adaptation to lower temperatures. Eggs are passively dispersed across the globe through container shipments of used tyres and wet plants [16, 17].

As a reaction to the dengue epidemics in Brazil, a national programme with the aim to eliminate *Ae. aegypti* (Programa para Erradicação do *Aedes aegypti*) was launched in 1996, and in 2002 it became the National Programme for Dengue Control (Programa Nacional de Controle de Dengue). The main goal of the programme is to fight dengue through integrated vector control strategies, including the use of larvicides [18]. Until 2008 the main larvicide used in the programme was the organophosphate temephos. Due to increasing resistance observed in several *Ae. aegypti* populations [19–21] temephos was replaced by the biological larvicide *Bacillus thuringiensis* svar. *israelensis* (*Bti*) in some municipalities in 2002. Then, in 2009, it was replaced by the insect growth regulators (i.e. chitin synthesis inhibitors), diflubenzuron and novaluron [19, 22] and finally, between 2014 and 2015, by pyriproxyfen, a juvenile hormone analogue [23]. Already in 2001, the health secretary of the city of Recife had decided to use *Bti* as the sole larvicide to fight *Ae. aegypti*. *Bti* equally targets *Ae. albopictus* larvae that share the same breeding sites with *Ae. aegypti* in many urban areas [24, 25] and, therefore, monitoring of *Bti* susceptibility to both *Aedes* species is needed.

In Switzerland, *Ae. albopictus* was found for the first time in the Canton of Ticino in the southernmost tip of the country in 2003 [13]; its surveillance has since been continuously expanded [26]. Today, the monitoring system consists of more than 1000 ovitraps that are analysed bi-weekly and the trapping data are used to coordinate targeted applications of insecticides [26]. In addition, public information campaigns are carried out in order to reduce breeding sites on private grounds. On public grounds, where larval breeding sites may not be removed (e.g. water drains), the authorities mainly apply *Bti* and diflubenzuron for larval control. In addition, focal spraying of permethrin to target adult mosquitoes is implemented if there is a risk of autochthonous transmission due to imported fever cases. Despite these efforts, *Ae. albopictus* has expanded its range in the Canton of Ticino over the last years [12, 26], requiring careful monitoring of the current insecticides’ efficacy.

*Bti* formulations are widely used and shown to be effective in controlling mosquitoes [27–29]. The toxicity of *Bti* against mosquito larvae is linked to crystals, produced during bacterial sporulation, that contain mainly four protoxins Cry11Aa, Cry4Aa, Cry4Ba and Cyt1Aa [30]. When ingested by mosquito larvae the crystals are dissolved in the alkaline milieu of the midgut and the released protoxins are then activated into toxins by gut proteases. The toxins bind to the receptors on the midgut cell membranes, leading to the formation of pores causing cell lysis, septicaemia and finally larval death [31, 32]. *Bti* toxins are highly active due to their synergistic effects to the target species, while showing low toxicity for other organisms due to their specificity [33]. *Bti* may be used in combination with *Lysinibacillus sphaericus*, another entomopathogen that also produces insecticidal crystals. In combination*,* the toxins from both bacteria display synergistic efficacy in a wide range of mosquito species, including *Aedes *spp. [29].

This study was part of a Brazilian-Swiss Joint Research Programme and aimed to examine whether the *Ae. albopictus* populations in the Swiss-Italian border region and in Recife are still fully susceptible to the insecticides currently applied despite their use over many years, in particular to *Bti* that has been widely employed in both study areas. While *Ae. albopictus* is the only potential vector of DENV, CHIKV and ZIKV in Switzerland, the main vector in Brazil is *Ae. aegypti* and, therefore, insecticide susceptibility assays were done for both *Aedes* species in Brazil. As there are no data available on adulticides in Switzerland, additional WHO insecticide susceptibility bioassays were carried out. In Ticino, Switzerland, a surveillance and control programme targets *Ae. albopictus*, such a programme does not exist in the neighbouring Lombardy region in Italy; hence mosquitoes were collected from both areas and their insecticide susceptibility compared.

## Methods

### *Aedes* susceptible reference colonies

Three *Aedes* colonies were used as susceptible reference colonies for all the compounds tested in this study: (i) Rockefeller, an international standard *Ae. aegypti* colony; (ii) RecL, an *Ae. aegypti* colony established from eggs collected from the Recife Metropolitan Region (RMR) in 1996 [34]; and (iii) RecLalb, an *Ae. albopictus* colony established from eggs collected from the same area as the RecL. The colonies were maintained in the insectary of the Instituto Aggeu Magalhães in Recife, Brazil as previously described [34]. Briefly, insects were maintained under controlled conditions at 26 ± 1 °C, 70% relative humidity and a 14:10 h light:dark photoperiod. Larvae were reared in de-chlorinated tap water and fed with cat food (Whiskas®, Brazil). Adults were fed on a 10% sucrose solution and females were provided with chicken blood twice per week.

### Establishment of *Aedes* field colonies

The field colonies were set up from eggs collected in the Canton of Ticino in southern Switzerland (TICINO), the Province of Como Lombardy in northern Italy (COMO) and from the Recife Metropolitan Region in Brazil, Sítio dos Pintos (SP) and Recife “field” (RF). The eggs for the TICINO and COMO colonies were provided by an already existing network of 280 ovitraps that were set across the Swiss-Italian border region [35]. The eggs were collected from the wooden slats in the ovitraps every other week between July and August 2013. To hatch out the eggs the slats were transferred to trays filled with de-chlorinated tap water. First-instar larvae were then split into equally sized batches, transferred to plastic trays and provided with TetraMin® fish food (Tetra, Melle, Germany). The larval trays were kept in a climate chamber (KBWF 720 E5.2, Binder GmbH, Tuttlingen, Germany) at 28 °C, 70% relative humidity and a 16:8 h light:dark photoperiod until pupation occurred. Adults that emerged from the pupae were transferred to a 30 × 30 × 30 cm Bugdorm-1® insect cage (Bugdorm, Taichung, Taiwan). Adults were allowed to mate and had access to water and 10% sucrose solution *ad libitum*. The founder populations of the TICINO and COMO colonies consisted of 520 (380 females and 140 males) and 610 (330 females, 280 males) adult mosquitoes, respectively. The females were blood-fed twice per week and their eggs collected on filter papers inside the cage to produce the test population. In Brazil, eggs were collected in 60 ovitraps distributed across Sítio dos Pintos (SP), a district of Recife city and used to establish the *Ae. albopictus* (SPalb) and *Ae. aegypti* (SPaeg) colonies, as described in Regis et al. [36]. Upon eclosion, larvae were maintained at the insectary of IAM-FIOCRUZ (Recife, Brazil) as described above. SPalb and SPaeg colonies were founded by 1774 (887 females and 887 males) and 3129 (1536 females and 1593 males) adult mosquitoes, respectively. Recife Field (RF), another *Ae. aegypti* colony representing 45 Recife districts was established from eggs sampled using ovitraps that were set according to the protocol previously described [37]. At least 1000 adults from these field collections were used to set up the RF colony. Bioassays were performed using larvae from the first (F_1_), the second (F_2_) or, in exceptional cases, also the third filial generation (F_3_).

### Larval bioassays

In the larval bioassays, the microbacteria *Bti,* individual *Bti *﻿toxi﻿ns﻿ and *L. sphaericus* as well as the chitin inhibiting diflubenzuron were tested against the above *Aedes* laboratory and field colonies (Table 1). *Bti* was prepared from the lyophilized reference powder IPS82 (Pasteur Institute, Paris, France), serotype H-14, as an aqueous suspensions at 5 g/l, and stored at -20 °C until use. In addition to *Bti*, individual *Bti* toxins, Cry11Aa and Cry4Ba, were produced with the *Bt* acrystalliferous strain 4Q2-81 that was transformed with plasmids carrying the respective protoxin genes [38]. Cry11Aa and Cry4Ba were chosen because they show the highest larval toxicity among the major protoxins of the crystal [39]. Spore-crystal biomass from each recombinant strain was produced and then lyophilized according to Barros et al. [40]. Similar to *Bti* an aqueous suspensions of *L. sphaericus* was prepared from lyophilized reference powder SPH88 (Pasteur Institute, Paris, France), serotype H5a5b strain 2362. Vectomax CG® (Valent Biosciences Corporation, Libertyville, IL, USA) is a commercial product available as water-soluble pouches containing a granular formulation that combines 4.5% *Bti* (serotype H-14, strain AM65-52) and 2.7% *L. sphaericus* (2362, serotype H5a5b, strain ABTS 1743) spores and insecticidal crystals as active ingredients (AIs). To prepare the stock suspension of 70 g/l (i.e. 5 g/l of AI) pouches (batch 179,654 N8) were incubated at 25 °C for 72 h in order to allow the release of crystals into the suspension. Aliquots of this suspension were then stored at -20 °C until use. Dose-response curves for *Bti*, Cry11Aa and Cry4Ba toxins, *L. sphaericus* and Vectomax® were estimated following the WHO guidelines for testing larvicides [41]. Briefly, batches of 20 third-instar larvae were exposed to serial dilutions of lyophilized spore-crystal powder in cups containing 100 ml bacterial suspensions in distilled water, without adding food. Five to seven concentrations for each compound were tested in each bioassay alongside a negative control group in three replicates. The negative control group was only exposed to distilled water. Each bioassay was repeated at least three times on different days. For *Bti*, Cry11Aa and Cry4Ba mortality rates were recorded after a 24 h exposure and for Vectomax® and *L. sphaericus* after a 48 h exposure time.

**Table 1:** *Aedes* spp. colonies and evaluated insecticides

Species	Colony	Source	Insecticidal compound						
			*Bti* H-14	Cry11Aa	Cry4Ba	Vectomax®	*L. sphaericus*	Diflubenzuron	Adulticides^a^
*Ae. albopictus*	RecLalb	Lab. Brazil	×	×	×	×	×	–	–
	TICINO	Field Switzerland	×	×	×	×	–	×	×
	COMO	Field Italy	×	×	×	×	–	–	×
	SPalb	Field Brazil	×	×	×	×	–	–	–
*Ae. aegypti*	Rockefeller	Lab. Brazil	×	×	×	–	–	×	–
	RecL	Lab. Brazil	×	–	–	–	–	–	–
	SPaeg	Field Brazil	×	×	×	–	–	–	–
	RF	Field Brazil	×	–	–	–	–	–	–

^a^Bendiocarb, DDT, malathion, permethrin (25:75 cis:trans ratio) and λ-cyhalothrin

In addition to the above larvicides, the efficacy of diflubenzuron was assessed against the TICINO colony because the compound is being used in the Ticino surveillance and control programme [26]. The efficacy of diflubenzuron to prevent adult emergence was assessed in third-instar larvae as it inhibits the production of chitin. For the bioassays diflubenzuron analytical standard powder (Sigma-Aldrich: St. Louis, MO, USA, code 45446) was dissolved in acetone to make a 0.3% (*w*/*v*) stock solution and aliquots were stored at -20 °C until use. The bioassays then followed the protocols described in Martins et al. [42]. Briefly, 8–12 concentrations, between 0.2 and 4.0 μg/l, were tested alongside a negative control containing a 0.2% acetone in water solution. To avoid starvation effects during the long term assay period food was added to the test cups. Larvae were exposed in 8 batches of 10, yielding 80 individuals at each diflubenzuron concentration and in the negative control. Any dead larva or pupa was removed from the bioassay cups every other day and adult emergence was observed up to 30 days. The assays were then repeated on different days up to four times (Additional file 1: Tables S1-S3).

The mortality and inhibition rates were the basis to estimate dose-response curves in order to predict the average concentrations, and 95% confidence intervals, at which 50% and 90% of the larval population would be killed (i.e. LC_50_ and LC_90_) or, in the case of diflubenzuron, prevented from reaching the adult stage (i.e. EI_50_ and EI_90_). The dose-response curves were estimated using generalised linear models with a binomial distribution and a “probit” link function. The models were computed using the statistical software IBM SPSS 10.0 for Windows.

On the basis of the estimated LC_50_s and LC_90_s a resistance ratio (RR) was calculated, where RR is the ratio between the LC for the test colony and the LC of the reference colony. For chemical insecticides, Mazzarri & Georghiou [43] proposed the following classifications: low resistance for an RR below 5, moderate resistance for an RR between 5 and 10, and high resistance for an RR above 10. However, for biological compounds such as *Bti*, RR values lower than 10-fold are considered as natural variations [19, 44].

### Adult bioassays

In addition to larvicides, the *Ae. albopictus* TICINO and COMO colonies sampled from the Swiss-Italian border region were also tested for their susceptibility against the four insecticide classes of WHO recommended adulticides. The insecticides evaluated were bendiocarb, dichlorodiphenyltrichloroethane (DDT), malathion, the non-alpha-cyanid pyrethroid permethrin (25:75 cis:trans ratio) and the alpha-cyanoid pyrethroid λ-cyhalothrin (Table 1). The pyrethroids were kindly provided by Syngenta Crop Protection (Basel, Switzerland), while the other insecticides were purchased as technical grades from Sigma-Aldrich. The bioassays were performed on females of the F_2_ generation raised from the field-sampled eggs following the WHO guidelines for testing adulticides [45]. Using a series of insecticide-impregnated filter papers, dose-response curves were estimated to determine the lethal dosage (LD) that would kill 50% (LD_50_) and 90% (LD_90_) of the TICINO and COMO colonies. The filter papers (Whatman no. 1) were impregnated with insecticide in acetone solutions mixed with silicon oil (Dow Corning 556 Silicon) according to the WHO test procedures [46]. The insecticide solutions were serial dilutions with five to six concentrations that would yield mortality rates between 0 and 100%. In the test, batches of 17–25 non-blood-fed *Ae. albopictus* females, aged 2–5 days were introduced into the exposure tubes lined with the insecticide-treated filter papers. The mosquitoes were exposed for 1 h, then gently blown back into the holding tube and provided with 10% sucrose solution. Following a 24 h recovery period, the numbers of dead and alive mosquitoes were recorded. Mosquitoes were considered to be alive if they were able to fly. Any knocked-down mosquito, with or without legs and wings, were considered moribund and were recorded as dead [45]. Tests were repeated aiming at 100 mosquitoes exposed per insecticide and concentration, including a negative control.

For the dose-response curves the 24 h mortality rates were the basis to estimate the dosage at which 50% and 90% of the adult population would be killed (i.e. LD_50_ and LD_90_). The dose-response curves were estimated using generalised linear models (GLM) with a binomial distribution and a “logit” link function, predicting mortality as a function of the log-transformed concentration. The models were computed in the freely available software package R, 3.3.2 [47] and the graph was produced with the R package “ggplot2” [48].

## Results

The susceptibility to *Bti* and its toxins was assessed for each *Aedes* population as they are all being exposed to *Bti* in the study areas*.* The *Ae. albopictus* populations from the Canton of Ticino in southern Switzerland, the Como area in northern Italy and Recife, Brazil were all still susceptible to *Bti*. The LC_50_ values were similar with a concentration of 0.015 mg/l, while the LC_90_ values varied between 0.030–0.036 mg/l (Table 2). RRs between the *Ae. albopictus* field colonies and RecLalb reference colony were all below two-fold, showing that the field populations remain fully susceptible to *Bti*. Likewise, the LC values for SPaeg and RF, the two *Ae. aegypti* populations from Brazil were close to those observed for the reference susceptible Rockefeller and the RecL colonies (Table 2). The LC values for *Bti* across *Ae. albopictus* and *Ae. aegypti* suggest that both species display a similar level of susceptibility to this agent.

**Table 2:** Toxicity of *Bacillus thuringiensis* svar. *israelensis* (IPS82) against third-instar *Aedes* spp. larvae

Species	Colony	Number	LC_50_ (95% CI)^a^	RR^b^	LC_90_ (95% CI)
*Ae. albopictus*	RecLalb^b^	1080	0.009 (0.008–0.011)	–	0.028 (0.023–0.037)
	TICINO	1440	0.015 (0.012–0.018)	1.7	0.036 (0.030–0.060)
	COMO	1120	0.015 (0.012–0.016)	1.7	0.030 (0.026–0.036)
	SPalb	1560	0.015 (0.011–0.020)	1.7	0.036 (0.027–0.098)
*Ae. aegypti*	Rockefeller^c^	1320	0.008 (0.007–0.009)	–	0.026 (0.021–0.036)
	RecL	1080	0.013 (0.011–0.015)	1.6	0.032 (0.027–0.039)
	SPaeg	1140	0.014 (0.012–0.016)	1.7	0.029 (0.025–0035)
	RF	1860	0.013 (0.012–0.016)	1.6	0.037 (0.030–0.050)

^a^Concentration (mg/l) that is lethal to 50% or 90% of the larvae over a 24 h exposure, mean and 95% confidence interval

^b^Resistance ratio (RR) between the LCs of the test colony and the reference colony

^c^Reference colony

In order to detect early development of resistance to individual *Bti* toxins, lyophilized powders containing Cry11Aa or Cry4Ba toxins were tested separately. Here, the LC_50_ values of Cry11Aa and Cry4Ba against the *Ae. albopictus* and *Ae. aegypti* field colonies were close to those found for the corresponding reference colonies (Table 3). The LC values of the selected Cry toxins were an order of magnitude higher than those of the overall *Bti* crystal, corroborating the *Bti* cocktail to be more effective than individual toxins.

**Table 3:** Toxicity of Cry11Aa and Cry4Ba against third-instar *Aedes* spp. larvae

		Cry11Aa			Cry4Ba		
Species	Colony	*n*	LC_50_ (95% CI)^a^	RR^b^	*n*	LC_50_ (95% CI)^a^	RR^c^
*Ae. albopictus*	RecLalb^b^	1500	0.410 (0.311–0.514)	–	1380	0.595 (0.431–0.787)	–
	TICINO	1020	0.539 (0.437–0.648)	1.3	1440	0.483 (0.213–0.839)	0.8
	COMO	1120	0.650 (0.517–0.798)	1.6	1060	0.782 (0.589–1.042)	1.3
	SPalb	1500	0.432 (0.335–0.530)	1.1	980	0.830 (0.622–1.095)	1.4
*Ae. aegypti*	Rockefeller^c^	1080	0.162 (0.121–0.210)	–	1120	0.331 (0.209–0.492)	–
	SPaeg	1140	0.266 (0.207–0.339)	1.6	1140	0.685 (0.482–0.969)	2.1

^a^Concentration (mg/l) that is lethal for 50% or 90% of the larvae over a 24 h exposure, mean and 95% confidence interval

^b^Resistance ratio (RR) between the LCs of the test colony and the reference colony

^c^Reference colony

The efficacy of *L. sphaericus*, another entomopathogenic bacterium, was tested against *Ae. albopictus* for which the susceptibility status has been poorly documented, in contrast to *Ae. aegypti* that is well known to be refractory. The reference powder SPH88 that contains crystals of the binary (Bin) toxin from the 2362 strain gave an LC_50_ of 0.084 mg/l and an LC_90_ of 0.336 mg/l in the RecLalb reference colony (Table 4).

**Table 4:** Lethal concentrations for Vectomax® and *Lysinibacillus sphaericus* (SPH88) against third-instar *Aedes albopictus* larvae

Larvicide	Colony	Number	LC_50_^a^ Mean (95% CI)	RR^b^	LC_90_^a^ Mean (95% CI)	RR^b^
Vectomax®	RecLalb	1140	0.087 (0.080–0.094)	–	0.163 (0.145–0.190)	–
	TICINO	1440	0.131 (0.118–0.144)	1.5	0.221 (0.194–0.228)	1.4
	COMO	1120	0.076 (0.069–0.083)	0.9	0.145 (0.130–0.169)	0.9
	SPalb	1260	0.092 (0.077–0.105)	1.1	0.191 (0.159–0.305)	1.2
*L*. *sphaericus*	RecLalb	1080	0.084 (0.070–0.099)	–	0.336 (0.239–0.630)	–

^a^Concentration (mg/l) that is lethal for 50% or 90% of larvae over a 48 h exposure, mean and 95% confidence limits

^b^Resistance ratio (RR) between the test colonies and the RecLalb reference colony

The activity of Vectomax®, a mixture of *Bti* and *L. sphaericus* crystals, was also investigated in order to evaluate if this combination of insecticidal components could be an effective alternative to control *Ae. albopictus*. Data from our evaluation showed similar LC values for TICINO, COMO and SPalb *Ae. albopictus* colonies (Table 4) and RRs below 2, suggesting Vectomax® to be effective.

The third control agent that was tested against immature mosquito stages was diflubenzuron, a compound used in Ticino to control *Ae. albopictus* [26] but neither in Recife nor Como; hence the efficacy of diflubenzuron was only evaluated against the TICINO colony. The EI_50_ preventing 50% larvae from developing into the adult stage was 0.376 mg/l (95% confidence interval; 95% CI: 0.289–0.462 mg/l) and the EI_90_ was 1.197 mg/l (95% CI: 1.033–1.448 mg/l). These concentrations were similar to those observed for the *Ae. aegypti* Rockefeller colony that was used as a reference. Here, the EI_50_ was 0.456 mg/l (95% CI: 0.352–0.549 mg/l) and the EI_90_ was 1.655 mg/l (95% CI: 1.322–2.249 mg/l).

**Fig. 1:**
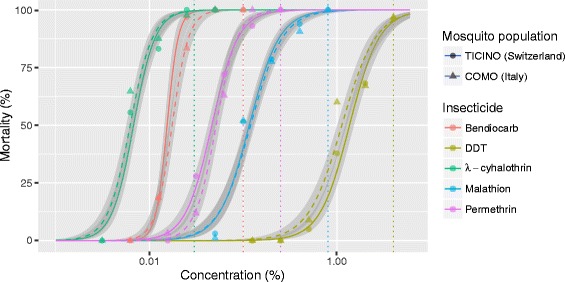
Dose-response effects of adulticides in *Aedes albopictus* from the Swiss-Italian border region. The curves show the estimated dose-response relationship between the 24 h mortality and the percentage insecticide on the filter paper in WHO insecticide susceptibility assays. The symbols represent the summaries of the actual measurements in the bioassays, while the *curves* are the predicted estimates of the mean and the shaded areas the 95% confidence intervals around the means. The vertical lines indicate the discriminating concentrations in *Ae. aegypti* for DDT, λ-cyhalothrin, malathion and permethrin, and in *Anopheles gambiae* for bendiocarb, as there are no discriminating concentrations established for *Ae. albopictus*

Finally, the two *Ae. albopictus* populations, COMO and TICINO from the Swiss-Italian border region were also tested against five insecticides, representing the four available classes of adulticides for which the susceptibility status was unknown. Among the five insecticides λ-cyhalothrin showed the lowest LC_50_, followed by bendiocarb, permethrin, malathion and DDT (Table 5). The two populations showed very similar dose-response profiles (Fig. 1). Although there are no diagnostic concentrations available for *Ae. albopictus*, adult mortalities after a 1 h exposure and 24 h holding period to bendiocarb, malathion, permethrin and λ-cyhalothrin suggest that both *Ae. albopictus* field populations are susceptible given that the mortality rates were close to 100% at the diagnostic concentration for other mosquito species (Fig. 1) and below those reported for permethrin and λ-cyhalothrin in previous studies of field populations [49, 50]. However, assuming *Ae. aegypti* to be a reference species, the COMO and TICINO colonies showed decreased sensitivity to DDT as mortality rates at the *Ae. aegypti* diagnostic concentration of 4% are estimated to be 96% for the TICINO and 96.9% for the COMO population, even at the extended exposure time of 1 h. Note that, according to WHO, the exposure time for *Ae. aegypti* against DDT would only be 30 min [51].

**Table 5:** Lethal concentrations for adulticides in *Aedes albopictus* from the Swiss-Italian border region

Insecticide	Population	Number^a^	LC_50_^b^ Mean (95% CI)	LC_90_^b^ Mean (95% CI)
Bendiocarb	TICINO	463	0.015 (0.014–0.017)	0.021 (0.019–0.024)
	COMO	470	0.017 (0.016–0.019)	0.027 (0.024–0.031)
DDT	TICINO	523	1.359 (1.220–1.514)	3.048 (2.557–3.635)
	COMO	470	1.126 (1.003–1.263)	2.807 (2.309–3.413)
λ-cyhalothrin	TICINO	440	0.007 (0.006–0.007)	0.012 (0.010–0.014)
	COMO	426	0.006 (0.006–0.007)	0.011 (0.010–0.013)
Malathion	TICINO	489	0.116 (0.104–0.128)	0.262 (0.222–0.310)
	COMO	486	0.120 (0.108–0.133)	0.284 (0.239–0.338)
Permethrin	TICINO	481	0.046 (0.042–0.051)	0.094 (0.081–0.110)
	COMO	430	0.051 (0.047–0.056)	0.092 (0.079–0.106)

^a^Total number of mosquitoes exposed across 5–6 concentrations

^b^Concentrations are expressed as % insecticide on the filter paper in the WHO insecticide susceptibility assay to kill 50% (LC_50_) and 90% (LC_90_) of the mosquito population over a 24 h holding period

The original data used in the statistical analysis are provided in the Additional file 1: Tables S1-S3.

## Discussion

In the absence of commercially available vaccines or treatments, dengue, chikungunya, Zika and other arbovirus transmissions may only be averted through vector control. However, vector control heavily relies on insecticides, raising concerns over the development of insecticide resistance besides adverse effects on the environment and human health [52]. Knowing the insecticide susceptibility status of a local mosquito population is, therefore, crucial [53]. Still, many programmes have been implemented without previously evaluating the susceptibility profiles of the target field populations to the intended control agents. In some cases, laboratory colonies have been used as surrogates to establish the susceptibility status, yet such colonies may underestimate the existence of resistance alleles in the field due to founder and bottle neck effects when maintaining laboratory colonies [44].

The biological larvicide *Bti* is known to be effective in reducing mosquito densities in control programmes and has a high toxicity to the target species without causing unwanted side-effects to the environment [28, 54]. The specificity of *Bti* is particularly important for the control of mosquito species that breed in ecologically sensitive areas where broad-spectrum insecticides may not be used. Likewise, in urban settings the control of day-active mosquito species like *Ae*. *albopictus* and *Ae. aegypti* by adulticides is critical because of human exposure to the insecticides. With the exception of one case in *Culex quinquefasciatus* in New York, USA [55], to our knowledge, no resistance to *Bti* has been reported from mosquito field populations [56–59] and decreased larval susceptibility to *Bti* is also rare [60–62]. Under laboratory conditions resistance has been found to single *Bti* toxins in selection experiments [63–65] but not to *Bti*. With regards to the case of *Bti *resistance reported from New York, it is inconclusive whether the observed resistance is linked to the application of *Bti* as there are neither data available from the pre-treatment period nor has the finding been confirmed in a follow up study.

Here, we performed larval bioassays with *Bti* reference powder IPS82 and two *Bti* toxins, Cry11Aa and Cry4Ba. Our study showed no increased tolerance in any of the *Aedes* populations and susceptibility was also similar between the intervention and the non-intervention areas in the Swiss-Italian border. Also, the results from the *Aedes* populations in Recife, Brazil suggest the exposure to *Bti* for several years had not selected for insecticide resistance. Comparing our results to the findings from other studies it appears that variations in *Bti* susceptibility in *Aedes* spp. are narrow [19, 61, 66, 67]. Likewise, our data show that *Ae. albopictus* and *Ae. aegypti* are equally susceptible to *Bti*, suggesting that the same application rates may be used where both species co-exist. This is an important finding since *Ae. albopictus* can be found in many urban environments together with *Ae. aegypti*, and presence of both species in these areas is being increasingly reported [24].

Tetreau et al. [65] stated that one of the main reasons why no resistance to *Bti* has yet been detected in the field is due to the synergistic effect of the individual toxins which may mask failure of individual toxins. Previous laboratory studies have shown that exposure to single *Bti* toxins selects for resistance but not when the toxins are combined [64, 67–69]. In this study the approach of Tetreau et al. [65] was followed and bioassays with two individual *Bti* toxins were performed with larvae in order to have a more sensitive assay that may detect early development of resistance. However, the mosquito test populations were still fully susceptible even to the individual Cry11Aa and Cry4Ba toxins. We, therefore, conclude that *Bti* treatments in both Ticino, Switzerland and Recife, Brazil have not exerted a selection pressure strong enough to cause a differential larval response to these individual toxins.

Like *Bti*, *L. sphaericus* is a naturally occurring soil bacterium that produces a larvicidal toxin [29]. The efficacy of *L. sphaericus* against *Ae. albopictus* has not been well investigated; and was also assessed here in order to account for the wide variations of *L. sphaericus* toxicity generally observed in this genus [70–72]. For *Ae. albopictus* we found LC values that were 8 to 13-fold higher than that reported for *C. quinquefasciatus* [73]. That is far better than the LC values for *Ae. aegypti*, which are 100 to 1000-fold higher and, for this reason, it is considered a refractory species [71]. When comparing LC values of both bacteria towards *Ae. albopictus*, *L. sphaericus* showed good activity since this species was only 11-fold less susceptible compared to *Bti*.

The biological larvicide Vectomax®, containing a mixture of *Bti* and *L. sphaericus* crystals, was also evaluated against *Ae. albopictus* since *L. sphaericus* activity can be enhanced by *Bti* [74]. Vectomax® combines *Bti*’s advantage of resistance-blocking together with *L. sphaericus*’ advantage of longer residuality in a single formulation and a broader spectrum of action. Our evaluation shows that Vectomax® is effective against *Ae. albopictus*, making it an alternative for the control of immature stages in our study areas. Conjugated products such as Vectomax® offer a mixture of 5 toxins that can target a wider range of medically important insect species, while showing a low risk of selection for resistance due to their complex mode of action. Other studies evaluating of Vectomax® in various environments have also shown its efficacy [75–77]. In conclusion, Vectomax® is a promising candidate to replace the actual microbial larvicides in our study areas.

Diflubenzuron showed to be another viable alternative to control *Ae. albopictus* larvae given the low inhibitory concentration found in the TICINO population, regardless of its history of utilisation in this area. Nevertheless, application of diflubenzuron needs careful assessment because it may harm non-target organisms.

In Ticino, the control of *Ae. albopictus* is mainly based on larval source reduction, either by removing breeding sites or by applying *Bti* or diflubenzuron if the larval sources cannot be removed [26]. Adulticides are rarely used. Only in exceptional cases is permethrin sprayed on vegetation when mosquito densities cause nuisance to residents in a confined area, or in surroundings from where symptomatic patients with arboviral disease have been reported [26]. Nevertheless, the susceptibility status of the Swiss *Ae. albopictus* population has never been investigated; and hence it is useful to know that permethrin shows good activity against the local *Ae. albopictus* population. While the results for permethrin, bendiocarb, λ-cyhalothrin and malathion suggest that the *Ae. albopictus* populations across the Swiss-Italian border region may be considered susceptible to these insecticides, there are some indications that the population shows decreased susceptibility to DDT, or perhaps even resistance. Alteration of susceptibility to DDT has also been recorded in *Ae. albopictus* in Thailand, Japan, Malaysia, Cameroon and the Central African Republic [49, 56, 78–80]; the underlying mechanisms remain unclear.

In Brazil, although the use of adulticides have decreased due to their toxicity to humans, resistance to most used compounds has already been widely documented in *Aedes* spp. populations [81, 82] and was not further investigated in the present study.

Although we made some inference about adulticide susceptibility in *Ae. albopictus*, we lack discriminating concentrations for this mosquito species. However, with its increasing importance for public health it would be helpful to have explicit discriminating concentrations also established for adulticides against *Ae. albopictus.*

In summary, the *Aedes* populations evaluated in this study were equally susceptible to the insecticides evaluated. The study implies that the currently applied mosquito larvicides in Ticino, southern Switzerland as well as in Recife, Brazil and adulticides in Ticino are still effective for the control of *Ae. albopictus* and *Ae. aegypti*. The larvicides tested have distinct modes of action and this feature is important to avoid the onset of resistance. Besides the use of insecticides, other strategies show promising results in decreasing vector densities and should be considered as part of integrated mosquito control programmes [83–87]. Highly productive artificial breeding sites are often found on private properties [83] and information campaigns encouraging the elimination of these water containers, alongside a correct use of biological insecticides, can significantly decrease the local mosquito population. The cost-effectiveness of such approaches and their long-term success should be evaluated when compared with conventional control methods [88]. Our results demonstrate the importance of research on the susceptibility status of mosquito populations to insecticides to prevent the spread of resistance in these important vectors of human diseases.

## Conclusions

Currently used larvicides (i.e. *Bti*, diflubenzuron) and adulticides (permethrin) used for mosquito control in the Ticino and Recife control programmes remain effective against the local *Ae. albopictus* and *Ae. aegypti* populations. The susceptibility profiles of the different mosquito populations were similar, despite distinct differences in the deployed interventions and geographical context. In addition, *Ae. albopictus* and *Ae. aegypti* display similar susceptibility levels to *Bti*, suggesting that this biolarvicide may target both species where they co-exist.

## Additional files

Additional file 1: Original data that were the basis for the statistical analysis. **Table S1.** Mortalities recorded in the bioassays with diflubenzuron. **Table S2.** Mortalities recorded in the bioassays with *Bti*, and individual *Bti* toxins. **Table S3.** Mortalities recorded in the bioassays with adulticides. (XLSX 122 kb)

## Abbreviations

95% CI: 95% confidence interval
*Bti*: *Bacillus thuringiensis* svar*. israelensis*
CHKV: Chikungunya virus
DDT: Dichlorodiphenyltrichloroethane
DENV: Dengue virus
EI: Emergence inhibition
LC: Lethal concentration
OP: Organophosphate
RR: Resistance ratio
ZIKV: Zika virus
